# Spermine oxidase promotes bile canalicular lumen formation through acrolein production

**DOI:** 10.1038/s41598-017-14929-1

**Published:** 2017-11-01

**Authors:** Takeshi Uemura, Tomokazu Takasaka, Kazuei Igarashi, Hiroshi Ikegaya

**Affiliations:** 1 Amine Pharma Research Institute, 1-8-15 Inohana, Chuo-ku, Chiba, 260-0856 Japan; 2 Department of Forensic Medicine, Graduate School of Medical Science, Kyoto Prefectural University of Medicine, 465 Kajii-cho, Kawaramachi-Hirokoji, Kamigyo-ku, Kyoto, 602-8566 Japan; 3 Graduate School of Pharmaceutical Sciences, Chiba University, 1-8-1 Inohana Chuo-ku, Chiba, 260-0856 Japan

## Abstract

Spermine oxidase (SMOX) catalyzes oxidation of spermine to generate spermidine, hydrogen peroxide (H_2_O_2_) and 3-aminopropanal, which is spontaneously converted to acrolein. SMOX is induced by a variety of stimuli including bacterial infection, polyamine analogues and acetaldehyde exposure. However, the physiological functions of SMOX are not yet fully understood. We investigated the physiological role of SMOX in liver cells using human hepatocellular carcinoma cell line HepG2. SMOX localized to the bile canalicular lumen, as determined by F-actin staining. Knockdown of SMOX reduced the formation of bile canalicular lumen. We also found that phospho-Akt (phosphorylated protein kinase B) was localized to canalicular lumen. Treatment with Akt inhibitor significantly reduced the formation of bile canalicular lumen. Acrolein scavenger also inhibited the formation of bile canalicular lumen. PTEN, phosphatase and tensin homolog and an inhibitor of Akt, was alkylated in a SMOX-dependent manner. Our results suggest that SMOX plays a central role in the formation of bile canalicular lumen in liver cells by activating Akt pathway through acrolein production.

## Introduction

Spermine oxidase (SMOX) is classified as a FAD (flavine adenine dinucleotide)-containing enzyme^1^ and catalyzes oxidative degradation of the polyamine spermine to produce spermidine^2^. SMOX is induced by a variety of stimuli including bacterial infections and oxidative stresses^3–5^. In the case of *Helicobacter pylori* infection, the induction of high SMOX activity increased reactive oxygen species dependent DNA damage^6^. Infection by *Bacteroides fragilis* induces SMOX activity and DNA damage in colon epithelial cells^4^. Furthermore, we have reported that acetaldehyde induced SMOX in the hepatocellular cell line HepG2 and increased acetaldehyde toxicity^5^.The cell damage caused by SMOX was mainly mediated by byproducts of spermine oxidation. Besides spermidine, SMOX also generates hydrogen peroxide (H_2_O_2_) and 3-aminopropanal, that is non-enzymatically converted to acrolein^7,8^. Acrolein is an unsaturated aldehyde and because of its high reactivity, the toxicity is 10 times higher than hydrogen peroxide^9^. Acrolein can react with amino acid residues in proteins, preferably cysteine, lysine and histidine, consequently modifying protein function and inducing apoptosis^10^ or tissue damage^11,12^.

For degradation of spermine, mammalian cells employ an alternative pathway in addition to SMOX that involves spermidine/spermine *N* ^1^-acetyltransferase (SSAT) and acetylpolyamine oxidase (PAOX)^13^. Spermine is acetylated by SSAT and then degraded by PAOX with less associated toxicity than that produced by the SMOX dependent pathway^14^ due to low production of acrolein. The existence of a less toxic, safer PAOX pathway suggests that SMOX has a physiological role other than regulation of spermine and spermidine contents in cells. Ever since it was identified and cloned by Wang *et al*.^15^, the role of SMOX has been actively investigated. However, research conducted so far has focused on the cytotoxic aspects of SMOX. In the present study, we investigated the physiological role of SMOX in the liver cells.

## Results

### SMOX localizes to bile canalicular lumen and is required for their formation in HepG2 cells

**Figure 1:**
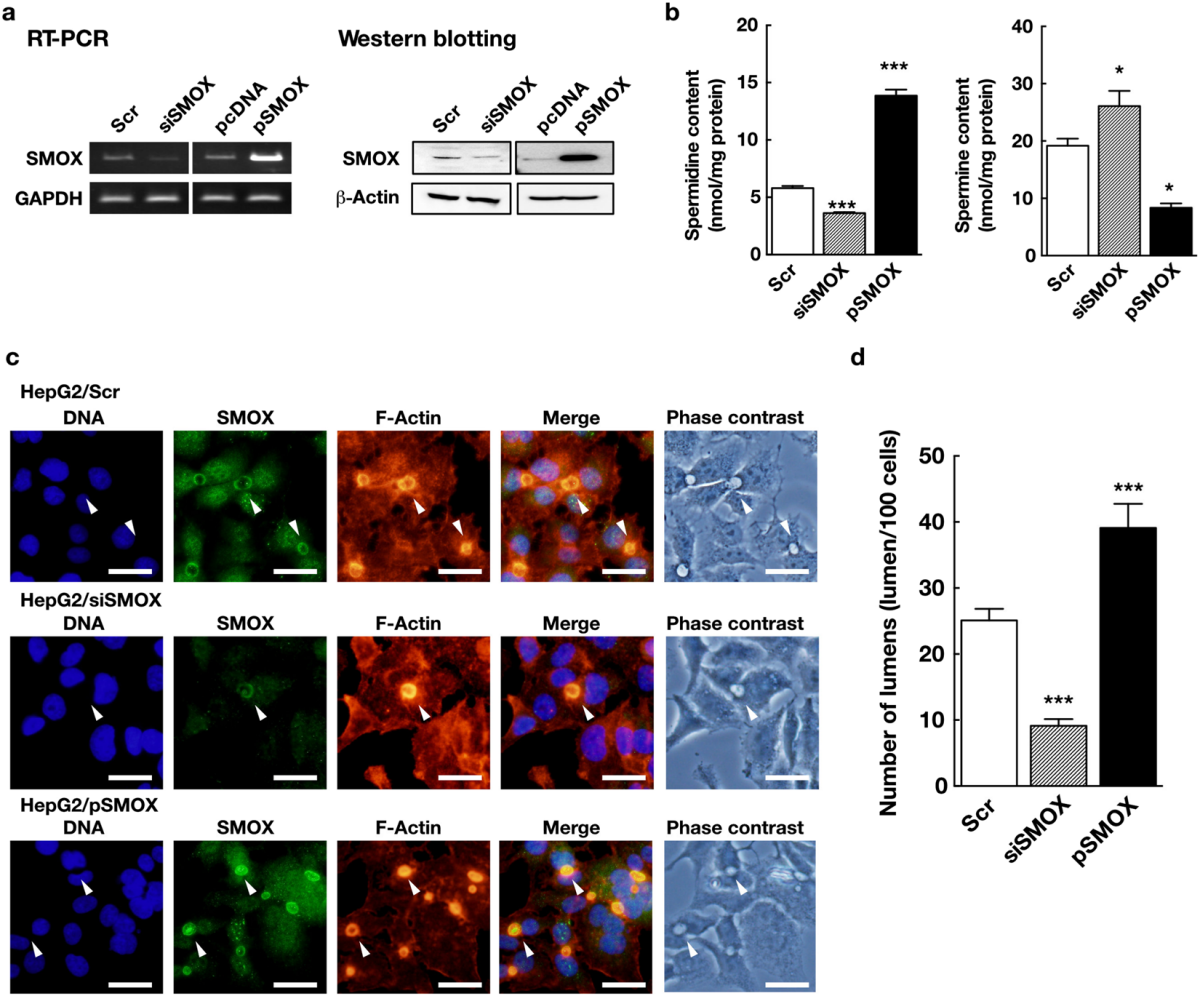
SMOX localizes to bile canalicular lumen and is required for their formation in HepG2 cells. (**a**) The levels of SMOX mRNA and protein in cells transfected with scrambled (negative control, Scr), SMOX-targeted siRNAs (siSMOX), pcDNA and pSMOX plasmids were determined by semi-quantitative PCR and western blotting as described in Materials and methods section. GAPDH and β-actin were used for loading control. Full-length blots/gels are presented in Supplementary Figure S1. (**b**) Spermidine and spermine contents in cells transfected with Scr (open column), siSMOX (dashed column) or pSMOX (filled column) were measured and expressed as nmol/mg protein. *p < 0.05, ***p < 0.005 against Scr. (**c**) HepG2 cells were transfected with Scr, siSMOX and pSMOX and cultured for 3 days on cover slips. SMOX and F-actin were stained as described in Materials and methods section. White arrowheads indicate bile canalicular lumens. Bar = 30 μm. (**d**) The numbers of bile canalicular lumen in cells 3 days after transfection with Scr (open column), siSMOX (dashed column) or pSMOX (filled column) were counted and expressed as the number of lumen per 100 cells. ***p < 0.005 against Scr.

To elucidate the physiological function of SMOX, we knockdown SMOX using siRNA as well as overexpressed by transfecting plasmid pSMOX^15^ encoding *SMOX* gene under CMV promoter. As shown in Fig. 1a, SMOX mRNA and protein levels were reduced to approximately 30% by siSMOX transfection. SMOX mRNA and protein were increased by pSMOX transfection (Fig. 1a). Knockdown of SMOX significantly reduced cellular spermidine content and increased spermine content (Fig. 1b), that indicated the oxidation of spermine to spermidine was decreased by SMOX knockdown. Overexpression of SMOX by pSMOX transfection increased spermidine content and decreased spermine content, that indicated the increased oxidation of spermine in transfected cells (Fig. 1b).

The subcellular distribution of SMOX in cells was analyzed next. As shown in Fig. 1c upper panels, SMOX was localized to the vacuole-like structure formed between two contacting cells as well as cytosols (Fig. 1c upper panels, white arrowheads). HepG2 are polarized cells that have the apical side at the space between cells to form bile canalicular lumen, which develop from small vacuoles between cells^16^. An accumulation of actin filaments is observed around the bile canaliculi, that represents the apical membrane domain^17^. Because of the accumulation of F-actin, the bile canalicular lumen can be stained with fluorophore-conjugated phalloidin^18^. We performed fluorescence staining of F-actin together with SMOX to identify the cellular localization of SMOX. Clear co-localization of SMOX and F-actin at vacuolar structure was observed, so we concluded that some portion of SMOX localized to the bile canalicular lumen (Fig. 1c).

We then investigated the role of SMOX at the bile canalicular lumen. As shown in Fig. 1c middle panels and Fig. 1d, knockdown of SMOX using siRNA significantly reduced the number of bile canalicular lumen. The bile canalicular lumen found in siSMOX transfected cells still express SMOX at bile canalicular lumen (Fig. 1c middle panels, white arrowheads). On the other hand, overexpression of SMOX significantly increased the number of bile canalicular lumen (Fig. 1c lower panels and Fig. 1d). The numbers of bile canalicular lumens per 100 cells were 25.1 ± 1.7 in scrambled RNA (Scr) transfected cells, 9.1 ± 1.0 in SMOX targeted siRNA (siSMOX) transfected cells and, 39.1 ± 3.7 in pSMOX transfected cells, respectively (Fig. 1d).

The effect of altered polyamine levels on the number of bile canalicular lumens was tested. When siSMOX transfected cells were cultured with 10 µM spermidine, the level of cellular spermidine was recovered to the level of Scr transfected cells (Supplementary Figure S2a). Culture with 10 µM spermine increased the spermine level in Scr transfected cells (Supplementary Figure S2b). In these cells, the numbers of bile canalicular lumen were not affected (Supplementary Figure S2c).

### SMOX and Phosphorylation of Akt are associated with lumen formation

**Figure 2:**
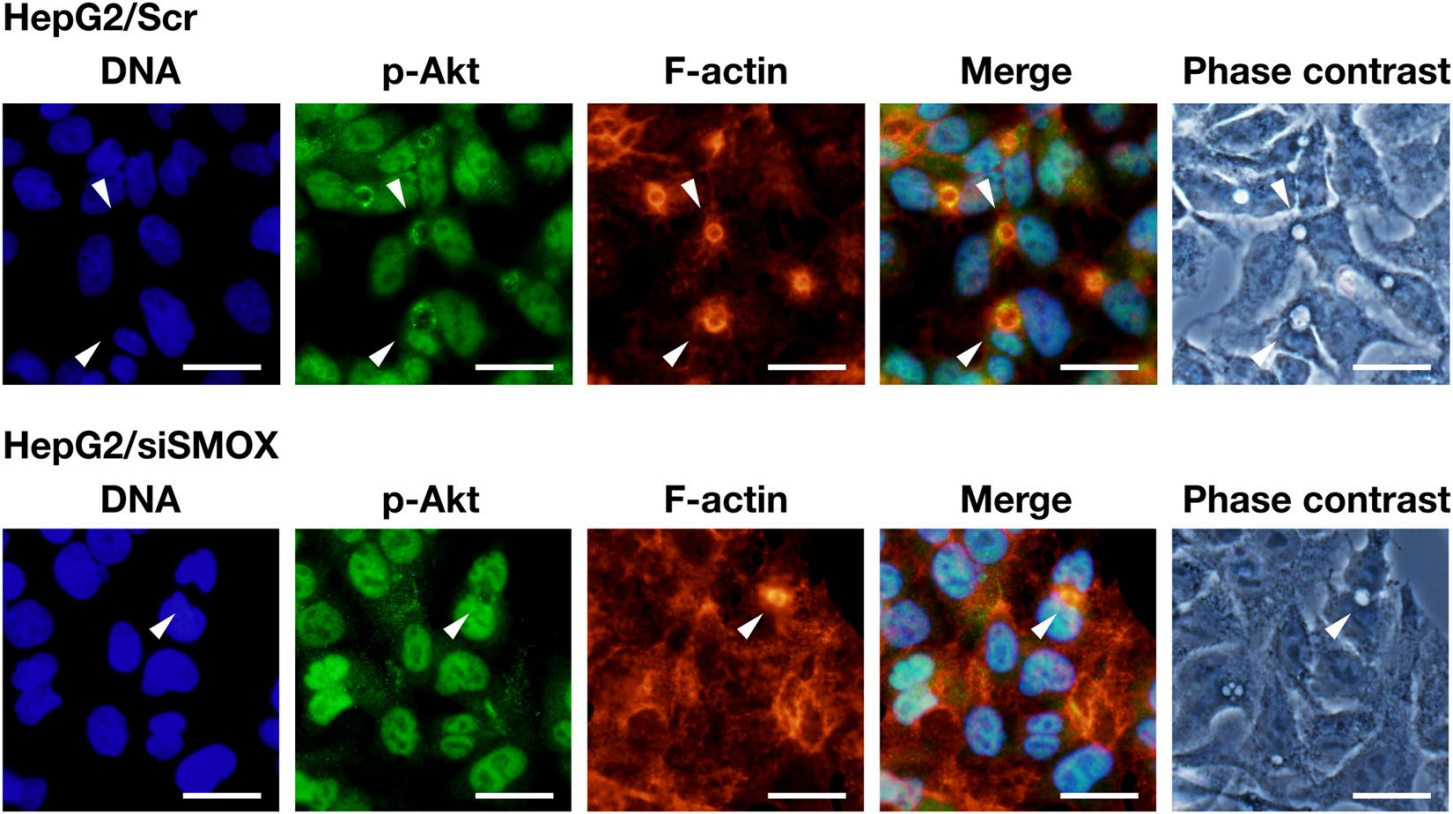
Phospho-Akt localizes to bile canalicular lumen. HepG2 cells were transfected with Scr and siSMOX RNAs, and cultured for 3 days on cover slips. Phospho-Akt (p-Akt) and F-actin were stained as described in Materials and methods section. White arrowheads indicate bile canalicular lumens. Bar = 30 μm.

The formation of bile canalicular lumen is accomplished by a dynamic reconstitution of plasma membrane and actin filaments^17^. For actin filament remodeling, phosphorylation of Akt, a protein kinase B, that activates the Akt pathway plays important roles^19^. We examined whether Akt phosphorylation was associated with lumen formation by determining the cellular distribution of phosphorylated Akt in both Scr and siSMOX transfected cells. As shown in Fig. 2, a significant portion of phospho-Akt was localized to the bile canalicular lumen in Scr transfected cells (white arrowheads) although the main localization of phospho-Akt was in nuclei. In cells transfected with siSMOX RNAs, where bile canalicular lumen formation was decreased, phospho-Akt was only localized to nuclei as cells had no bile canalicular lumen (Fig. 2, lower panels). There was no bile canalicular lumen without phospho-Akt signal (Fig. 2, lower panels, white arrowhead). The result suggests that phosphorylation of Akt is associated with SMOX dependent bile canalicular lumen formation.

**Figure 3:**
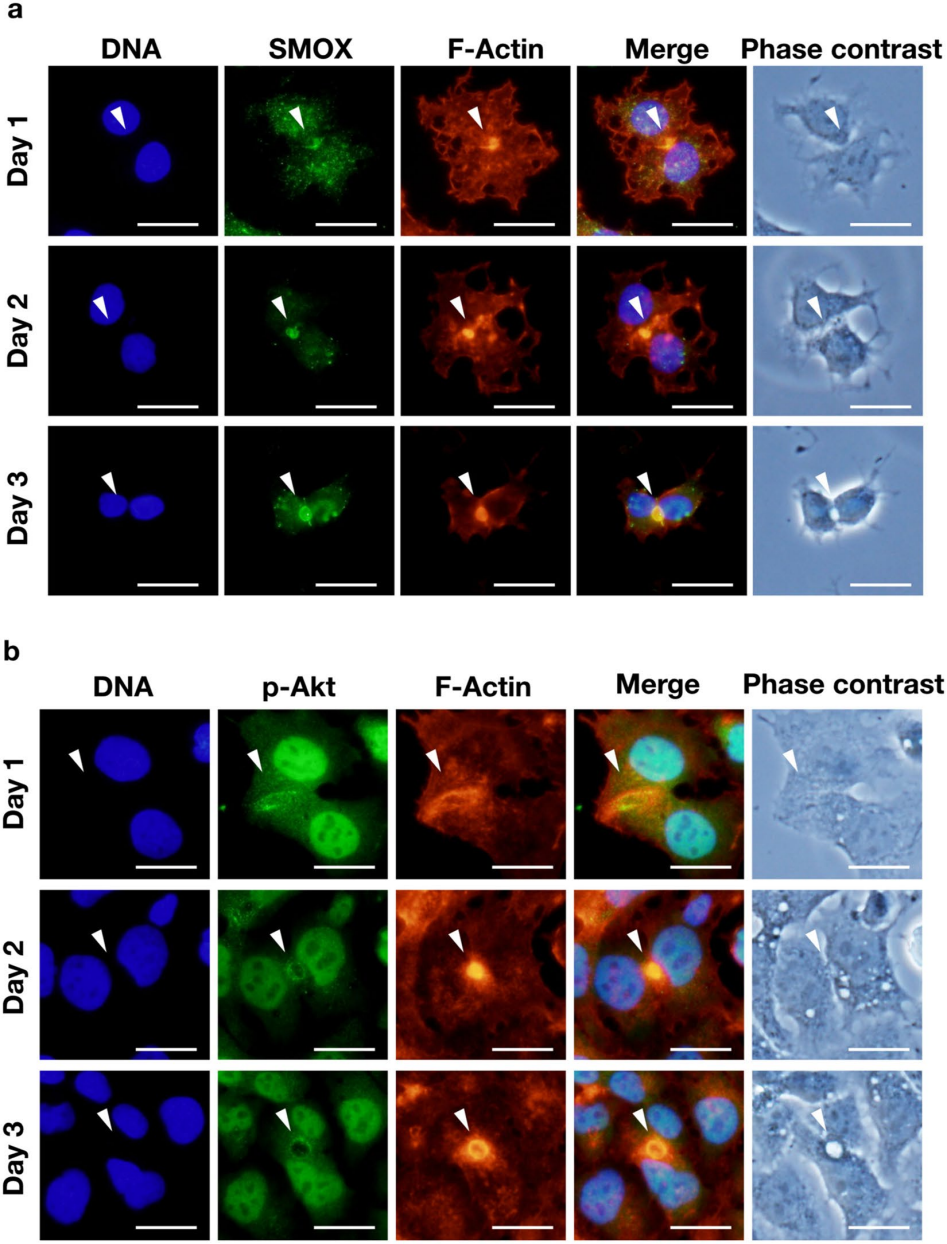
SMOX, phospho-Akt and F-actin associate during bile canalicular lumen formation. HepG2 cells were cultured on the cover slip and fixed on day 1, 2 and 3. SMOX, phospho-Akt and F-actin were stained and observed as described in Materials and methods section. White arrowheads indicate bile canalicular lumens. Bar = 30 μm.

We next examined the time-dependent change of SMOX, F-actin and phospho-Akt distribution in cells during bile canalicular lumen formation. As shown in Fig. 3a, SMOX and F-actin started to accumulate at the locus between cells that become bile canalicular lumen afterwards, before lumen was formed on day 1 (Fig. 3a, upper panels). The signal of SMOX and F-actin increased as lumen structure grew on day 2 (Fig. 3a, middle panels). When lumen was formed on day 3, SMOX and F-actin remained on the lumen (Fig. 3a, bottom panels). The similar patterns were observed in the case of phospho-Akt (Fig. 3b). The signal of phospho-Akt together with actin grew during lumen formation. Time-dependent change of SMOX and phospho-Akt was not observed in SMOX knockdown cells as they did not develop the bile canalicular lumen (Supplementary Figure S3). These results suggest that SMOX and Akt pathway play significant roles in the bile canalicular lumen formation.

### The Akt pathway together with acrolein is essential for lumen formation

**Figure 4:**
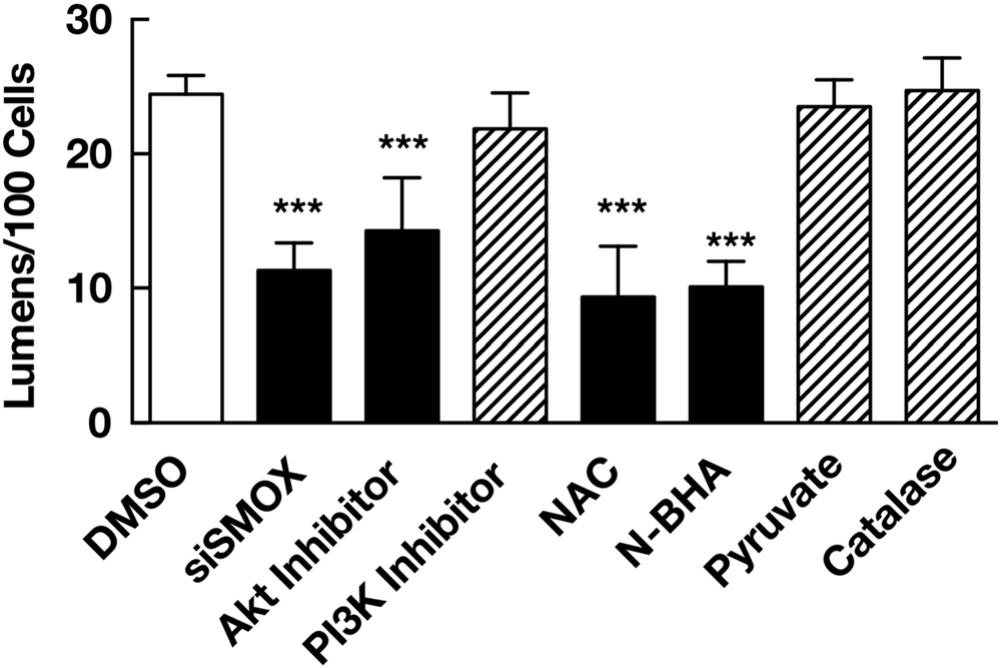
SMOX, Akt pathway and acrolein are required for bile canalicular lumen formation. Cells (5 × 10^5^ cells for each treatments) were transfected with siSMOX or treated with 10 µM Akt inhibitor 1L6-hydroxymethyl-chiro-inositol-2-(R)-2-O-methyl-3-O-octadecyl-*sn*-glycerocarbonate, 5 nM PI3K inhibitor 17β-hydroxywortmannin, 1 mM *N*-acetylcysteine (NAC), 1 mM *N*-benzylhydroxylamine (*N*-BHA), 4 mM pyruvate or 10^4^ units/mL catalase for 3 days. The number of bile canalicular lumen were counted and expressed as numbers of lumen/100 cells. Values are expressed as mean + SD of three independent experiments. ***p < 0.001 against DMSO (dimethylsulfoxide).

To examine the importance of the Akt pathway in bile canalicular lumen formation, the effect of Akt inhibitor 1L6-hydroxymethyl-chiro-inositol-2-(R)-2-O-methyl-3-O-octadecyl-*sn*-glycerocarbonate on bile canalicular lumen formation was tested. As shown in Fig. 4, Akt inhibitor treatment significantly reduced the number of bile canalicular lumens (14.3 ± 4.0/100 cells) compared to vehicle treated cells (24.4 ± 1.4/100 cells). The reduction of lumen number by Akt inhibitor was comparable to that by siSMOX transfection (11.3 ± 2.1/100 cells). Since the PI3K pathway regulates the Akt pathway, the effect of PI3K inhibitor 17β-hydroxywortmannin (HWT) on bile canalicular lumen formation was also tested. The HWT treatment did not affect the number of lumen formed (21.9 ± 2.7/100 cells).

We next investigated the role of acrolein, a product of spermine oxidation by SMOX in bile canalicular lumen formation by treating cells with acrolein scavengers. Both *N*-acetylcysteine (NAC) and *N*-benzylhydroxylamine (*N*-BHA), known acrolein scavengers^7,8^, significantly decreased the number of bile canalicular lumen (9.3 ± 3.8/100 cells for NAC and 10.1 ± 1.9/100 cells for *N*-BHA, respectively). Since NAC is reported to scavenge not only acrolein but also hydrogen peroxide, also produced by oxidation of spermine^20^, we tested the specificity of NAC and *N*-BHA for acrolein. Both NAC and *N*-BHA reduced acrolein cytotoxicity whereas only NAC but *N*-BHA reduced the cytotoxicity of hydrogen peroxide (Supplementary Figure S4). These results indicated that *N*-BHA neutralized acrolein but not hydrogen peroxide. We also tested the effect of pyruvate and catalase, both of which neutralize hydrogen peroxide^9^. As shown in Fig. 4, pyruvate and catalase did not decrease the number of lumens (23.5 ± 2.0/100 cells for pyruvate and 24.7 ± 2.4/100 cells for catalase, respectively). These results indicate that Akt pathway and acrolein, but not hydrogen peroxide, are required for bile canalicular lumen formation. The effects of exogenous acrolein and H_2_O_2_ on the number of bile canalicular lumens were then tested. In contrast to SMOX overexpression that increased endogenous acrolein production from spermine, exogenous acrolein reduced the number of bile canalicular lumens in both Scr and siSMOX transfected cells (Supplementaru Figure S4). The result suggests that exogenous acrolein disrupts the polarized distribution of acrolein generated by SMOX in cells and inhibits the formation of bile canalicular lumen. Exogenous H_2_O_2_ did not affect the number of bile canalicular lumens (Supplementaru Figure S4).

### PTEN and β-actin are alkylated by SMOX dependent mechanism

**Figure 5:**
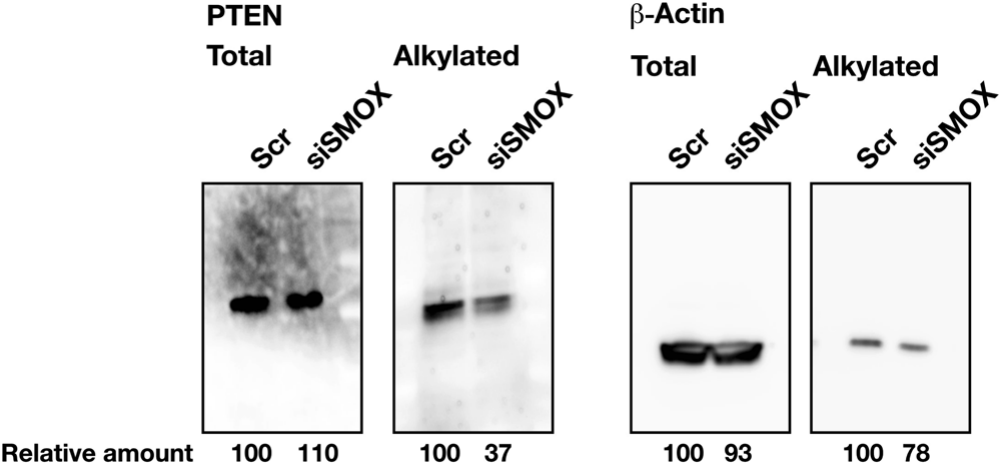
PTEN and β-actin are alkylated in a SMOX-dependent manner. Three million cells were transfected with Scr and siSMOX RNAs, cultured for 3 days. Alkylated PTEN and β-actin were biotin-tagged and detected as described in Materials and methods section. Total PTEN and β-actin levels were measured by western blot analysis using 40 μg of cell extract. Full-length blots are presented in Supplementary Figure S6.

The results obtained so far led us to hypothesize that acrolein produced by SMOX could impact Akt phosphorylation and bile canalicular lumen formation. As Covey *et al*. reported^21^, acrolein can alkylate and inactivate PTEN, phosphatase and tensin homolog and an inhibitor of Akt^22^. Accordingly, we measured the level of alkylated PTEN in SMOX knockdown cells. As shown in Fig. 5, siSMOX transfection reduced the amount of alkylated PTEN whereas total PTEN was not affected. We also measured the level of β-actin alkylation in siSMOX transfected cells. Transfection of siSMOX slightly decreased alkylated β-actin whereas total β-actin level was not affected (Fig. 5). These results clearly show that PTEN and β-actin are alkylated in an SMOX-dependent manner.

## Discussion

In this report, we investigated the physiological functions of SMOX in liver cells using human hepatocellular carcinoma derived HepG2 cells. We found that SMOX localized to the bile canalicular lumen, which was strongly stained with Alexa Fluor 546 conjugated phalloidin (Fig. 1c). Knockdown of SMOX significantly reduced the number of bile canalicular lumens (Fig. 1c,d). The existing bile canalicular lumen in siSMOX transfected cells still stained with SMOX (Fig. 1c middle panels, white arrowheads) and there was no bile canalicular lumen without SMOX. This result suggests that SMOX plays crucial roles in the formation of bile canalicular lumen. Overexpression of SMOX significantly increased the number of bile canalicular lumen (Fig. 1c,d). This result also suggests that SMOX plays important roles in the formation of bile canalicular lumen. Knockdown of SMOX reduced spermidine content and increased spermine content, and overexpression of SMOX increased spermidine and decreased spermidine, indicating that SMOX plays a significant role in the regulation of cellular polyamine levels (Fig. 1b). Recovery of spermidine level in siSMOX transfected cells or increased spermine level in Scr transfected cells by exogenous polyamines did not affect the number of bile canalicular lumens (Supplementary Figure S2). This result indicates that alteration of cellular polyamines by SMOX is not a direct effector for the bile canalicular lumen formation.

The localization of phospho-Akt at bile canalicular lumen suggested that the accumulation of F-actin at the lumen site was regulated by actin remodeling activated by the Akt pathway^19^. Time-dependent observation of SMOX, phospho-Akt and F-actin distribution revealed that SMOX, phospho-Akt and F-actin coordinately involved in the bile canalicular lumen formation (Fig. 3). SMOX, phospho-Akt and F-actin started to co-localize to the border of two cells before the bile canalicular lumen was formed. The amount of both SMOX, phospho-Akt and F-actin increased during lumen formation (Fig. 3). In the SMOX knockdown cells, this accumulation of phospho-Akt and F-actin was not observed (Supplementary Figure S3) as the bile canalicular lumen was not formed. These results suggested that SMOX was required for actin accumulation that is necessary for bile canalicular lumen formation^17^.

Akt inhibitor treatment significantly decreased the number of bile canalicular lumens formed relative to the comparable number of SMOX knockdown cells (Fig. 4). PI3K inhibitor did not decrease the number of bile canalicular lumen. This result suggests that SMOX is involved in the Akt dependent and PI3K independent pathway for bile canalicular lumen formation. Covey *et al*. reported that acrolein alkylated and inactivated PTEN to activate the Akt pathway^21^. Together with this report, our results suggest that SMOX stimulated bile canalicular lumen formation by producing acrolein that could inactivate PTEN, and then activated the Akt pathway and actin remodeling. This hypothesis was tested using acrolein scavenger *N*-acetylcysteine (NAC) and *N*-benzylhydroxylamine (*N*-BHA)^7,8^. Both NAC and *N*-BHA significantly reduced lumen number whereas neutralization of hydrogen peroxide, that is also produced by SMOX at the same molecular ratio of acrolein, by pyruvate and catalase did not affect the number of lumens (Fig. 4). The specificity of NAC as acrolein scavenger was tested by cell growth experiment (Supplementary Figure S4). NAC reduced the cytotoxicity of acrolein as well as H_2_O_2_. *N*-BHA reduced only cytotoxicity of acrolein (Supplementary Figure S4). There are some reports concerning the specificity of *N*-BHA as an acrolein scavenger^23,24^. Together with these reports, our results indicate that *N*-BHA specifically neutralizes acrolein and NAC neutralizes both acrolein and H_2_O_2_. The results strongly suggest that acrolein but not hydrogen peroxide produced by SMOX was important for bile canalicular lumen formation. The hypothesis was further confirmed by measuring alkylated PTEN level in SMOX knockdown cells. Knockdown of SMOX significantly reduced the modified PTEN level (Fig. 5). This result indicated that SMOX contributed to the activation of Akt pathway by inactivating PTEN via acrolein production. We also found that β-actin was SMOX-dependently alkylated. The alkylation of β-actin might be also involved in the remodeling of actin filaments.

Exogenous acrolein inhibited bile canalicular lumen formation (Supplementary Figure S5). It was suggested that exogenous acrolein disrupted the polarized distribution of acrolein and alkylated PTEN. For bile canalicular lumen formation, the localized of SMOX to the apical side of cells is very critical. Since acrolein is highly reactive^1^, only adjacent protein to SMOX will be alkylated by acrolein. This polarized distribution of acrolein dependent modification can contribute to the oriented actin remodeling to form bile canalicular lumen. Disruption of the polarity of acrolein modification by exogenous acrolein can inhibit bile canalicular lumen formation. Exogenous H_2_O_2_ did not affect the number of bile canalicular lumens. This result supports our idea that acrolein is a major contributor for the formation of bile canalicular lumen.

**Figure 6:**
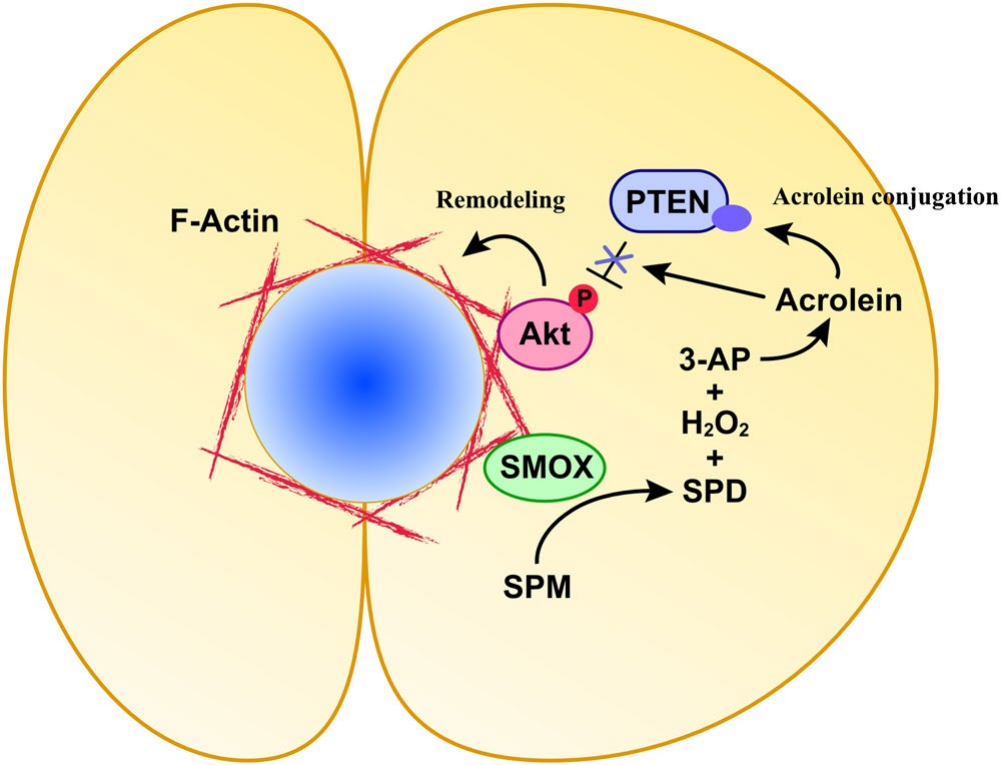
The role of SMOX in the bile canalicular lumen formation. SMOX catalyzes the oxidative degradation of spermine to spermidine at the apical membrane where two cells are in contact with each other. Oxidation of spermine by SMOX produces 3-aminopropanal (3-AP) which is spontaneously converted to acrolein. Acrolein alkylate and inactivate adjacent PTEN, causing phosphorylation and activation of Akt. Activation of Akt stimulates F-actin remodeling and induces bile canalicular lumen formation. SPD, spermidine, SPM, spermine, 3-AP, 3-aminopropanal, PTEN, phosphatase and tensin homolog.

The role of SMOX in bile canalicular lumen formation, that is suggested by our results, is summarized and depicted in Fig. 6. SMOX localizes to the site that will form bile canalicular lumen and generates 3-aminopropanal, which is spontaneously converted to acrolein, and hydrogen peroxide by oxidation of spermine to spermidine. Generation of acrolein alkylates and inactivates adjacent PTEN that causes the activation of Akt. Phosphorylated, activated Akt stimulates actin remodeling for canalicular lumen formation. The mechanism of SMOX localization to the lumen is still to be clarified and investigation is underway.

The bile canaliculus is a 1 to 2 μm wide tube that collects bile secreted from hepatocytes and eventually merges to form hepatic bile ducts^25^. The deficiency or destruction of bile canaliculi leads to failure of bile secretion that causes severe disorders including primary billiary cirrhosis^26,27^. Our results reveal that SMOX and polyamine metabolism plays a critical role in the formation of bile canaliculi. SMOX catalyzes the oxidative degradation of spermine to spermidine and generates acrolein and hydrogen peroxide as byproducts^1^. Acrolein is a highly reactive aldehyde and toxicity is approximately ten times higher than hydrogen peroxide^9^. Previous research on acrolein and SMOX so far focused on the cytotoxicity^28^. Our results provide the first evidence that indicates SMOX and acrolein play a pivotal role in the physiological function of cells.

## Materials and Methods

### Cell culture and transfection

The human hepatocyte carcinoma-derived HepG2 cells, provided by the RIKEN BRC through the National Bio-Resource Project of the MEXT, Japan, were cultured in DMEM medium supplemented with 50 U/ml streptomycin, 100 U/ml penicillin G and 10% fetal calf serum at 37 °C in an atmosphere of 5% CO_2_. When cells were cultured in the presence of spermidine or spermine, 1 mM aminoguanidine (Tokyo Chemical Industry, Tokyo, Japan) was added to the media to inhibit degradation of spermidine and spermine by amine oxidase in calf serum. For cell growth analysis, cells were inoculated at 2 × 10^5^ cells/well to 6-well plate (BD Falcon, the size of growing area is 9.6 cm^2^/well) and cultured in the presence of 20 µM acrolein (Tokyo Chemical Industry, Tokyo, Japan) or 150 µM H_2_O_2_ (nacalai tesque, Japan) with 1 mM *N*-acetylcysteine (NAC, Sigma) or 1 mM *N*-benzylhydroxylamine (*N*-BHA, Sigma) for 5 days and viable cells were counted. The viable cell number was counted under a microscope in the presence of 0.25% trypan blue (nacalai tesque, Japan).

The Stealth RNAi targeting human SMOX and negative control scrambled RNAi were purchased from Invitrogen. Sequences of RNAs targeting human SMOX are 5′-UCAUUGAGAUGUACCGAGACCUCUU-3′ and 5′-AAGAGGUCUCGGUACAUCUCAAUGA-3′, 5′-CGCAAGUACUAUUCCACCATT-3′ and 5′-UGGUGGAAUAGUACUUGCGGT-3′. The plasmid pSMOX that encoding *SMOX* gene under CMV promoter and its vector pcDNA 3.1^15^ were kindly provided by Dr. Robert A. Casero, the Sidney Kimmel Comprehensive Cancer Center at Johns Hopkins University, Baltimore, Maryland. One million cells were transfected with 100 pmol each of RNAs or 2 µg of plasmids using Lipofectamine 2000 reagent (Invitrogen) according to the manufacturer’s protocol in Opti-MEM reduced serum medium (GIBCO).

### Indirect Immunofluorescence Microscopy

Cells were cultured on cover slips, washed with phosphate buffered saline (PBS) and fixed in 2% paraformaldehyde for 15 minutes at 37 °C. Cells were soaked in acetone for 15 seconds at -20 °C. Cells were treated with 1% bovine serum albumin in PBS and incubated with primary antibody for 16 hours at 4 °C. Primary antibodies used are anti-SMOX antibody raised against human SMOX protein (Proteintech, USA) and anti-phospho-Akt (abcam). Cells were washed 10 times with the same buffer and incubated with fluorophore labeled secondary antibody for 16 hours at 4 °C. Cells were washed 10 times and mounted in Prolong Gold Mounting Solution containing DAPI for staining of nuclei (Clontech). For staining of F-actin, cells were incubated with Alexa Fluor 546 conjugated phalloidin (Molecular Probes) for 30 min at room temperature. Fluorescence was visualized using an OLYMPUS IX73 microscope equipped with a DP73 digital camera (OLYMPUS). For counting of lumens, cells were stained with DAPI and Alexa Fluor 546 conjugated phalloidin and the numbers of cells and lumens were counted manually under the microscope. The lumen count was expressed as the number of lumen per 100 cells.

### Measurement of polyamine contents

Polyamine contents in cells were determined using the method described by Igarashi K *et al*.^29^. Cells were washed three times with ice-cold phosphate buffered saline (PBS), homogenized in 10 mM Tris-HCl, pH 8.0 and incubated in 0.2 M perchloric acid at 70 °C for 30 min. After centrifugation, supernatants were collected and resulting pellet were homogenized and incubated in 0.2 M perchloric acid at 70 °C for 30 min. After centrifugation, supernatants were combined and analyzed using a SHIMADZU HPLC system with TSKgel Polyaminepak column (TOSOH, Japan). Protein contents in cells were determined with a BCA protein assay kit (Nacalai tesque) using bovine serum albumin as a standard.

### Semi-quantitative PCR analysis

Levels of mRNA of SMOX and glyceraldehyde 3-phosphate dehydrogenase (GAPDH) were measured using SuperPrep^TM^ Cell Lysis & RT Kit (TOYOBO) according to the manufacturer’s protocol using primer sets of SMOX-F (5′-CGGACGGTATGCAAAGTT-3′) and SMOX-R (5′-TGTCGTGATTGTGGTCGC-3′) for SMOX, and GAPDH-F (5′-TGGTATCGTGGAAGGACTCGTGGAAGGACTCATGAC-3′) and GAPDH-R (5′-AGAGTCCAGTGAGCTTCCCGTTCAGC-3′) for GAPDH.

### Western blotting

Cells were washed with PBS and lysed in 10 mM Tris-HCl, pH 8.0 containing 10 μg/ml aprotinin, 500 μM sodium orthovanidate, 10 μg/ml phenylmethylsulfonyl fluoride. Protein was separated on a 10% polyacrylamide gel and transferred electrophoretically to a Hybound-C PVDF membrane (Amersham, Arlington Heights, IL). Blots were blocked in 5% nonfat dry milk in tris-buffered saline containing 0.1% Tween 20 (TBS-T) for 30 min at room temperature. SMOX, PTEN and β-actin were detected by an ECL Western Blotting Detection System (GE Healthcare) using anti-SMOX (Proteintech, USA), anti-PTEN (Millipore) and anti-β-actin (SANTA CRUZ BIOTECHNOLOGY) as primary antibodies.

### Treatment of cells with Akt and PI3 kinase inhibitors, acrolein and H_2_O_2_ scavengers

Cells (5 × 10^5^ cells) were inoculated on the coverslips and cultured for 3 days with 10 µM Akt inhibitor 1L6-hydroxymethyl-chiro-inositol-2-(R)-2-O-methyl-3-O-octadecyl-*sn*-glycerocarbonate (Calbiochem), 5 nM PI3K inhibitor 17β-hydroxywortmannin (Merck Millipore), 1 mM *N*-acetylcysteine (NAC, Sigma), 1 mM *N*-benzylhydroxylamine (N-BHA, Sigma), 4 mM pyruvate (nacalai tesque, Japan) or 10^4^ units/mL catalase (Sigma) for 3 days. The number of bile canalicular lumen were counted as described above and expressed as numbers of lumen/100 cells.

### Determination of modified PTEN

HepG2 cells were transfected with NC or siSMOX and cultured for 3 days. Alkylated PTEN was determined by biotin-conjugated maleimide tagging method described by Covey *et al*.^21^. In brief, cells were frozen at −80 °C for 15 min and incubated in 1 ml of extraction buffer (50 mM NaHPO4, pH 7.0, 1 mM EDTA, 10 mM *N*-ethyl maleimide, 10 mM iodoacetic acid, 1% Triton X-100, 5 mM NaF and proteinase inhibitor cocktail) at 25 °C for 1 h and washed with the same buffer. After addition of SDS to a final concentration of 1% and incubating at 25 °C for 2 h in the dark, proteins were precipitated with 10% of trichloroacetic acid (TCA). The precipitate was washed with acetone and incubated at 50 °C for 30 min in reducing buffer (50 mM Hepes-NaOH, pH 7.7, 1 mM EDTA, 2% SDS and 4 mM dithiothreitol). Proteins were then biotinylated with 1 mM biotin conjugated to polyethylene oxide-maleimide (Thermo Scientific) for 30 min at 50 °C. Proteins were incubated with immobilized neutravidine beads (Thermo Scientific) in 1 ml PBS containing 0.4% v/v Tween 20 for 16 h at 4 °C. After washing twice with the same buffer, maleimido-biotinylated proteins were analyzed by western blotting.

### Statistical analysis

Statistical analysis was performed using GraphPad Prism version 6.0d for Mac, GraphPad Software, La Jolla California USA, www.graphpad.com. Differences between two groups were compared using Student’s t-test. For comparison of multiple groups, one-way ANOVA followed by Bonferroni’s multiple comparisons test was used. A *p*-value < 0.05 was considered statistically significant.

The datasets generated during and/or analysed during the current study are available from the corresponding author on reasonable request.

## Electronic supplementary material

Supplementary Figures

## References

[1] Pegg AE (2013). Toxicity of polyamines and their metabolic products. Chem. Res. Toxicol..

[2] Cervelli M (2016). Stability of spermine oxidase to thermal and chemical denaturation: comparison with bovine serum amine oxidase. Amino Acids.

[3] Chaturvedi R (2013). Spermine oxidase is a regulator of macrophage host response to Helicobacter pylori: enhancement of antimicrobial nitric oxide generation by depletion of spermine. Amino Acids.

[4] Goodwin AC (2011). Polyamine catabolism contributes to enterotoxigenic Bacteroides fragilis-induced colon tumorigenesis. Proc Natl Acad Sci USA.

[5] Uemura, T. *et al*. Acetaldehyde-induced cytotoxicity involves induction of spermine oxidase at the transcriptional level. *Toxicology* doi:10.1016/j.tox.2013.05.008 (2013). 10.1016/j.tox.2013.05.008 23707493

[6] Chaturvedi R (2004). Induction of polyamine oxidase 1 by Helicobacter pylori causes macrophage apoptosis by hydrogen peroxide release and mitochondrial membrane depolarization. J Biol Chem.

[7] Saiki R (2009). Intense correlation between brain infarction and protein-conjugated acrolein. Stroke.

[8] Saiki R (2011). Brain infarction correlates more closely with acrolein than with reactive oxygen species. Biochem Biophys Res Commun.

[9] Yoshida M (2009). Acrolein toxicity: Comparison with reactive oxygen species. Biochem Biophys Res Commun.

[10] Nakamura M (2013). Inactivation of GAPDH as one mechanism of acrolein toxicity. Biochem Biophys Res Commun.

[11] Hirose T (2015). Increase in acrolein-conjugated immunoglobulins in saliva from patients with primary Sjögren’s syndrome. Clin. Chim. Acta.

[12] Uemura T (2017). Activation of MMP-9 activity by acrolein in saliva from patients with primary Sjögren’s syndrome and its mechanism. Int. J. Biochem. Cell Biol..

[13] Casero RA, Pegg AE (1993). Spermidine/spermine N1-acetyltransferase–the turning point in polyamine metabolism. FASEB J..

[14] Uemura T (2016). Decrease in acrolein toxicity based on the decline of polyamine oxidases. Int. J. Biochem. Cell Biol..

[15] Wang Y (2001). Cloning and characterization of a human polyamine oxidase that is inducible by polyamine analogue exposure. Cancer Res.

[16] Zaal KJ, Kok JW, Sormunen R, Eskelinen S, Hoekstra D (1994). Intracellular sites involved in the biogenesis of bile canaliculi in hepatic cells. Eur. J. Cell Biol..

[17] Zegers MM, Zaal KJ, van IJzendoorn SC, Klappe K, Hoekstra D (1998). Actin filaments and microtubules are involved in different membrane traffic pathways that transport sphingolipids to the apical surface of polarized HepG2 cells. Mol. Biol. Cell.

[18] Herrema H (2006). Rho kinase, myosin-II, and p42/44 MAPK control extracellular matrix-mediated apical bile canalicular lumen morphogenesis in HepG2 cells. Mol. Biol. Cell.

[19] Xue G, Hemmings BA (2013). PKB/Akt-dependent regulation of cell motility. J. Natl. Cancer Inst..

[20] Capone C (2013). A role for spermine oxidase as a mediator of reactive oxygen species production in HIV-Tat-induced neuronal toxicity. Free Radic. Biol. Med..

[21] Covey TM, Edes K, Coombs GS, Virshup DM, Fitzpatrick FA (2010). Alkylation of the tumor suppressor PTEN activates Akt and β-catenin signaling: a mechanism linking inflammation and oxidative stress with cancer. PLoS ONE.

[22] Matsuda S, Kobayashi M, Kitagishi Y (2013). Roles for PI3K/AKT/PTEN Pathway in Cell Signaling of Nonalcoholic Fatty Liver Disease. ISRN Endocrinol.

[23] Koyama R, Mizuta R (2017). Acrolein scavengers, cysteamine and N-benzylhydroxylamine, reduces the mouse liver damage after acetaminophen overdose. J. Vet. Med. Sci..

[24] Grigsby J, Betts B, Vidro-Kotchan E, Culbert R, Tsin A (2012). A possible role of acrolein in diabetic retinopathy: involvement of a VEGF/TGFβ signaling pathway of the retinal pigment epithelium in hyperglycemia. Curr. Eye Res..

[25] Esteller A (2008). Physiology of bile secretion. World J. Gastroenterol..

[26] Stapelbroek JM, van Erpecum KJ, Klomp LWJ, Houwen RHJ (2010). Liver disease associated with canalicular transport defects: current and future therapies. J. Hepatol..

[27] Saxena R, Hytiroglou P, Thung SN, Theise ND (2002). Destruction of canals of Hering in primary biliary cirrhosis. Hum. Pathol..

[28] Cervelli M, Amendola R, Polticelli F, Mariottini P (2012). Spermine oxidase: ten years after. Amino Acids.

[29] Igarashi K (1986). Formation of a compensatory polyamine by Escherichia coli polyamine-requiring mutants during growth in the absence of polyamines. J Bacteriol.

