# Supplementary material for: Spermine oxidase promotes bile canalicular lumen formation through acrolein production
Source: Sci Rep. 2017 Nov 1;7:14841. doi: 10.1038/s41598-017-14929-1 (PMC5665972; doi:10.1038/s41598-017-14929-1)
Supplement: Supplementary file 1 — Supplementary Figures [file 41598_2017_14929_MOESM1_ESM.pdf]

Supplementary Figures

Manuscript title: Spermine oxidase promotes bile canalicular lumen formation through acrolein production

Author list:

Takeshi Uemura[1, 2*], Tomokazu Takasaka[2], Kazuei Igarashi[1, 3] and Hiroshi Ikegaya[2]

[1]Amine Pharma Research Institute, 1-8-15 Inohana, Chuo-ku, Chiba 260-0856, Japan

[2]Department of Forensic Medicine, Graduate School of Medical Science, Kyoto Prefectural University of Medicine, 465 Kajii-cho, Kawaramachi-Hirokoji, Kamigyo-ku, Kyoto 602-8566, Japan

[3]Graduate School of Pharmaceutical Sciences, Chiba University, 1-8-1 Inohana Chuo-ku, Chiba 260-0856, Japan

Supplementaly Figure S1

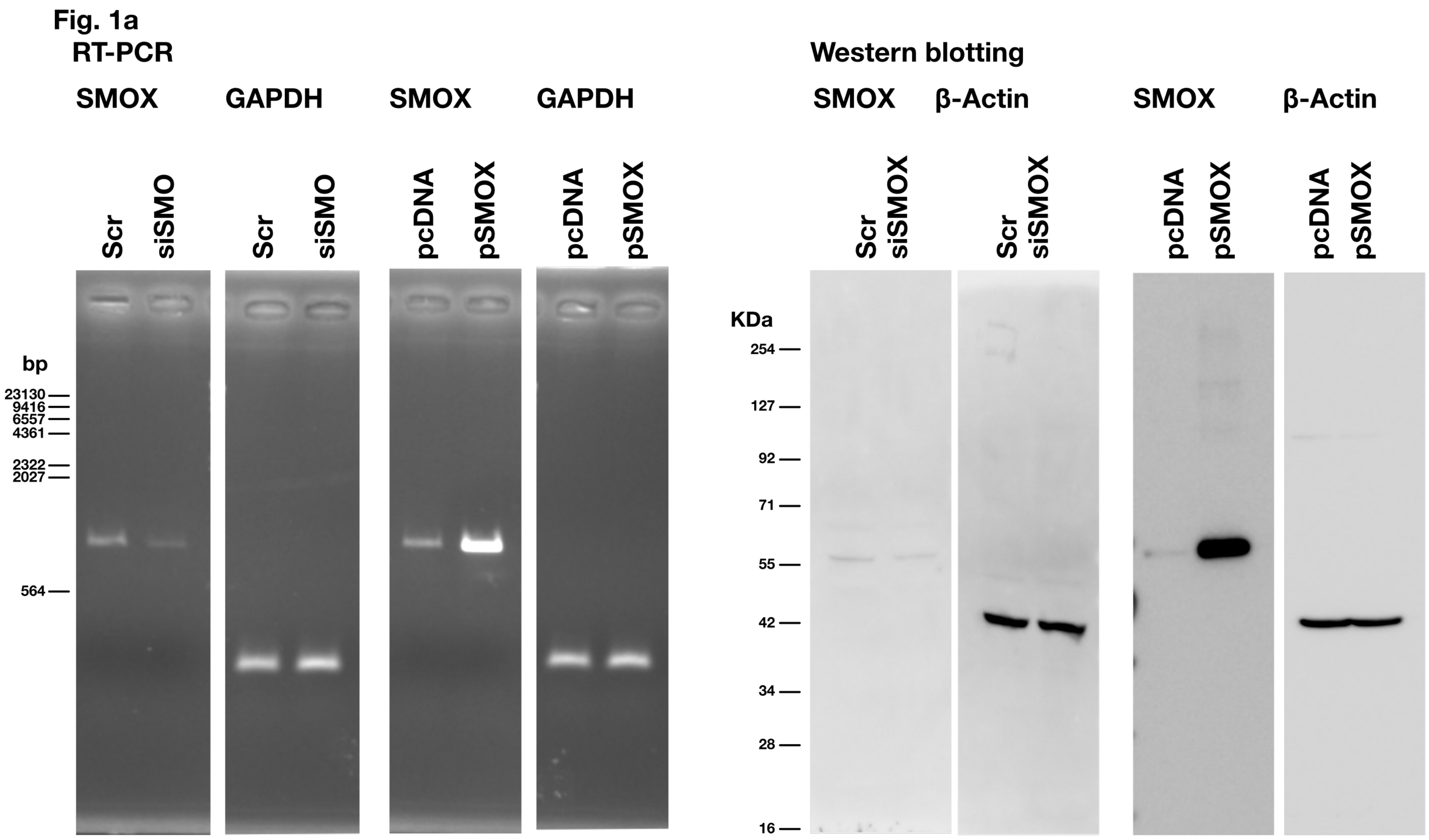

Supplementary Figure S1. Full-length blots/gels for Fig. 1a are presented. The levels of SMOX mRNA and protein were determined by semi-quantitative PCR and western blotting as described in Materials and methods section. GAPDH and β-actin were used for loading control.

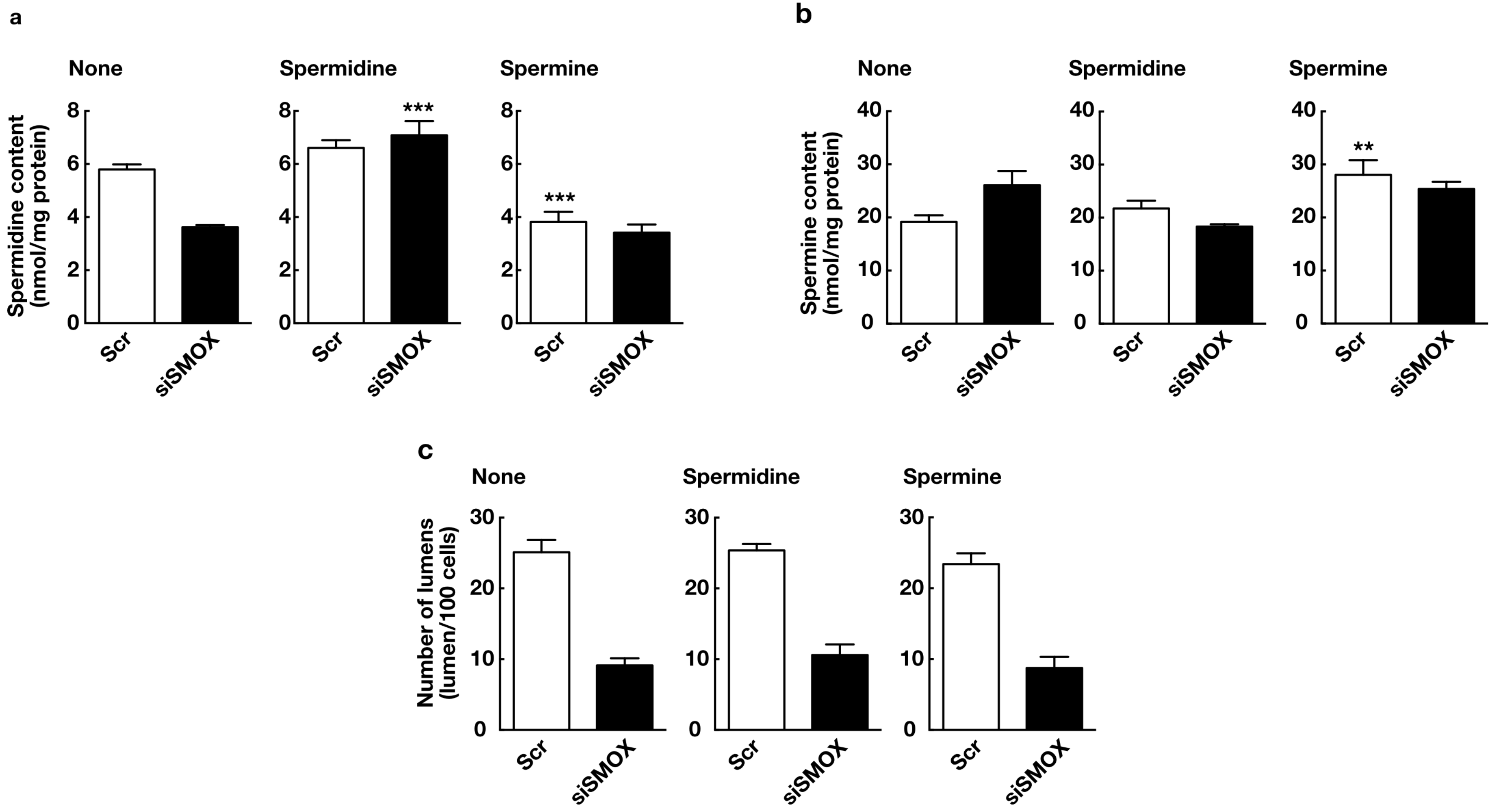

Supplementary Figure S2. Spermidine (a) and spermine (b) contents in cells transfected with Scr (open column) or siSMOX (filled column) RNAs cultured with 10 μM each of spermidine or spermine were measured as described in Materials and Method and expressed as nmol/mg protein. (c) The numbers of bile canalicular lumen in cells 3 days after transfection with Scr (open column) or siSMOX (filled column) RNAs cultured with 10 μM each of spermidine or spermine were counted and expressed as the number of lumen per 100 cells.**, $p < 0.01$, ***, $p < 0.005$ against None.

Supplementaly Figure S3

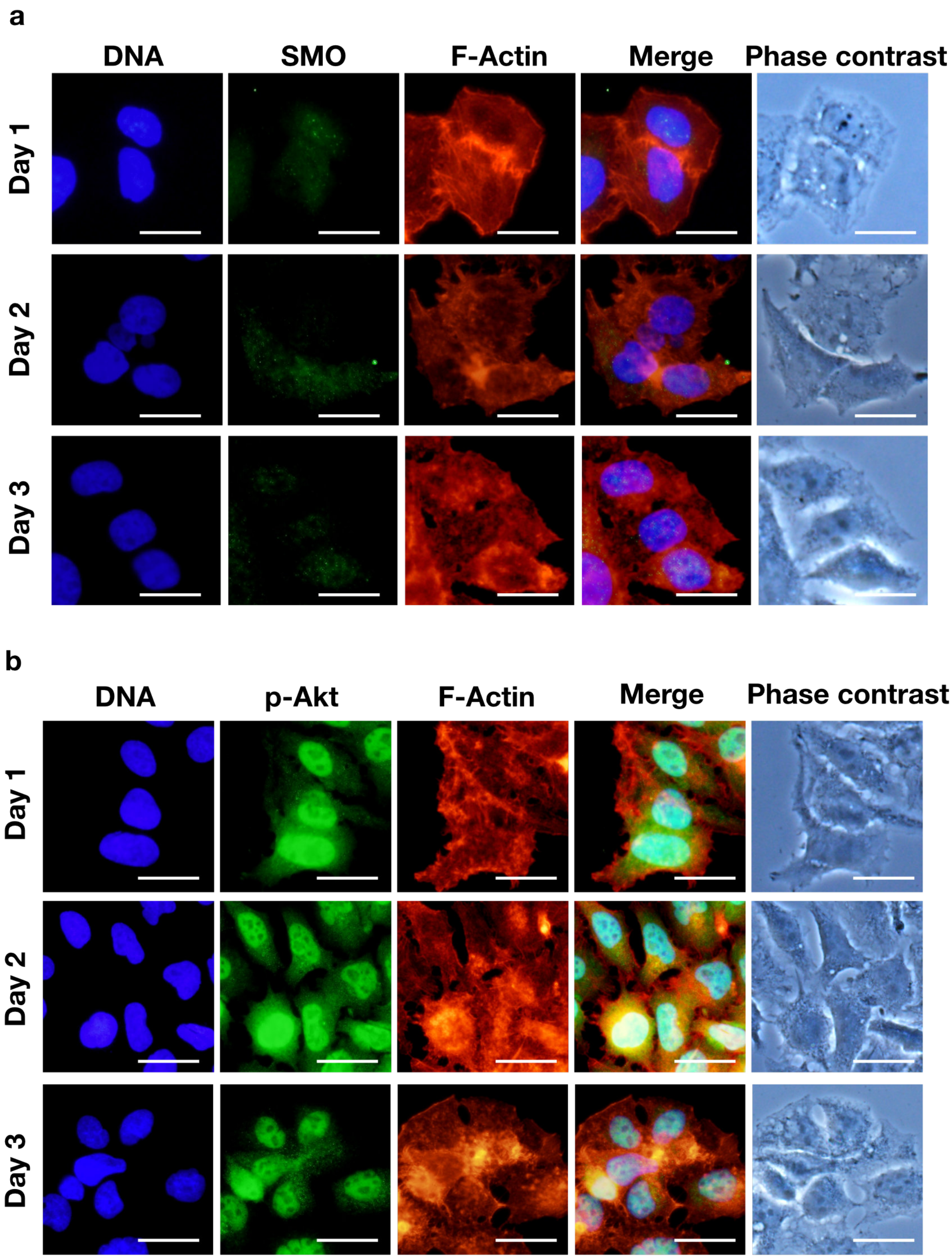

Supplementary Figure S3. HepG2 cells transfected with siSMOX were cultured on the cover slip and fixed on day 1, 2 and 3. DNA, SMOX (a), phospho-Akt (p-Akt) (b) and F-actin were stained and observed as described in Materials and methods section. Bar = 30 μm.

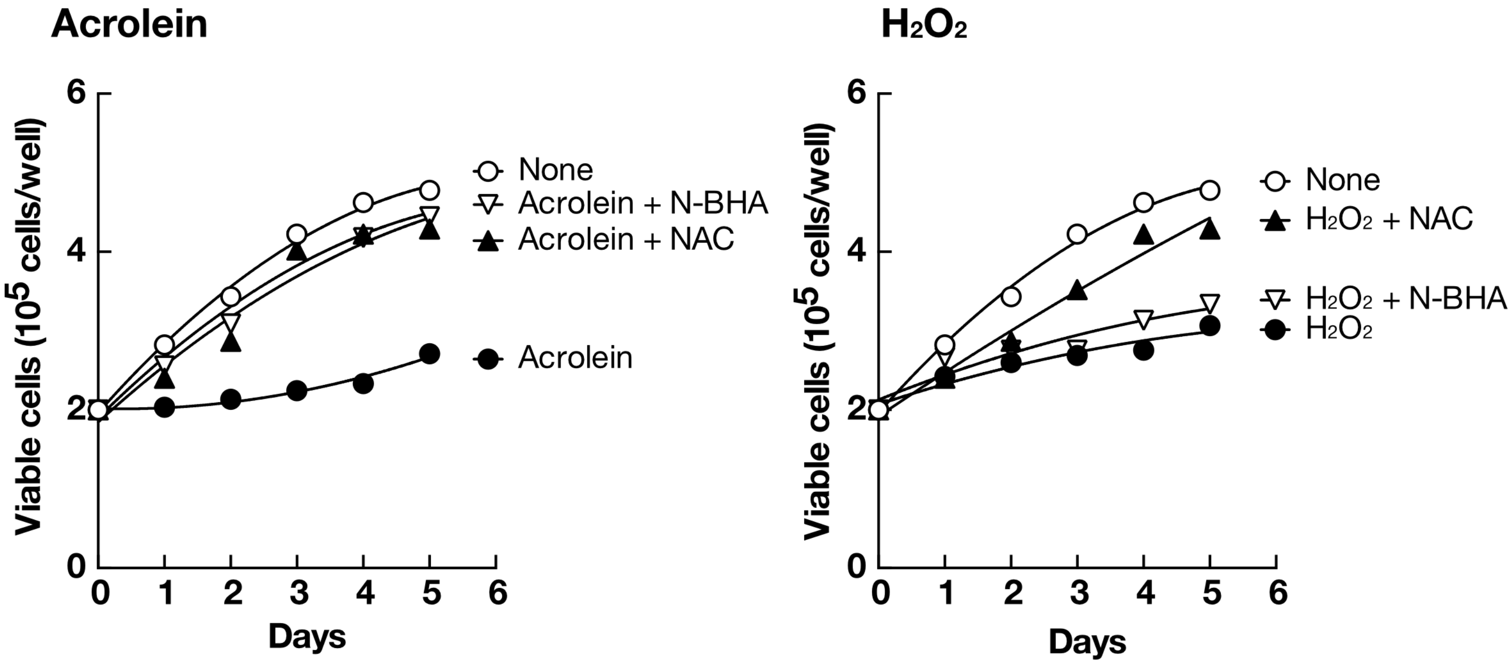

Supplementary Figure S4. HepG2 cells were inoculated at 2×10^5 cells/well in a 6-well plate (the size of growing area is 9.6 cm^2 /well) and cultured in the presence of 20 μM acrolein or 150 μM H_2O_2 with 1 mM *N*-acetylcysteine (NAC) or 1 mM *N*-benzylhydroxylamine (N-BHA) for 5 days and viable cells were counted. The viable cell number was counted under a microscope in the presence of 0.25% trypan blue.

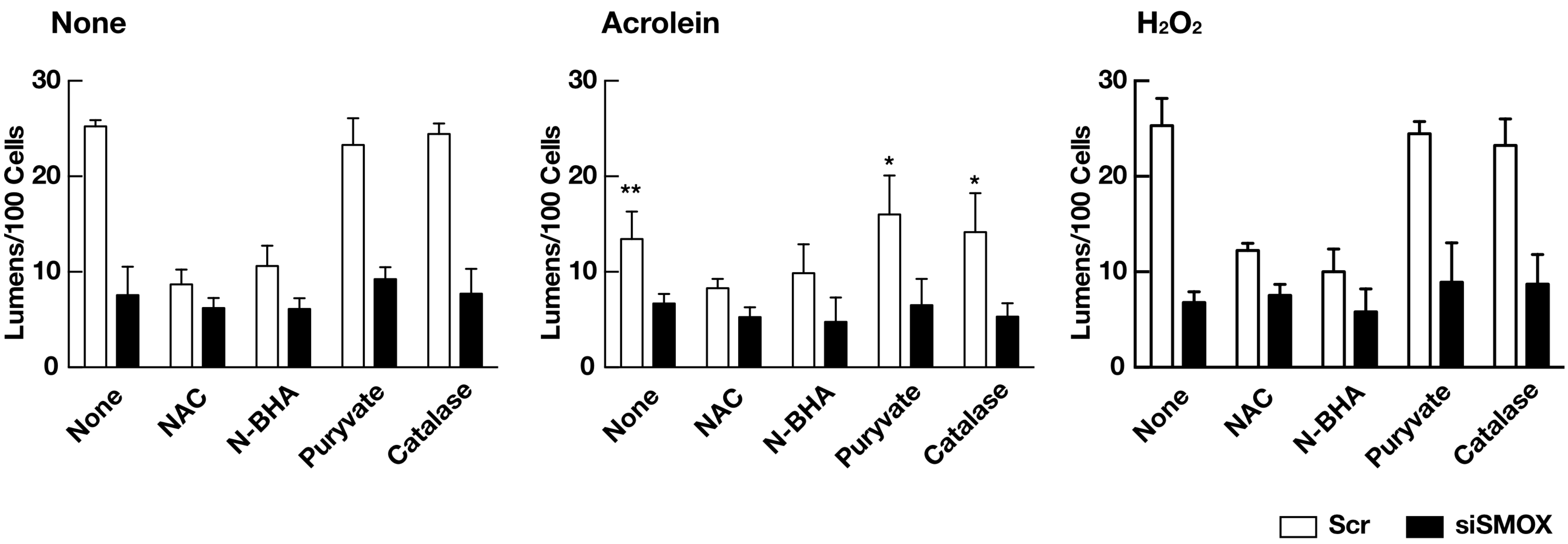

Supplementary Figure S5. HepG2 cells transfected with Scr (open column) or siSMOX (filled column) RNAs (5×10^5 cells for each treatments) were 1 mM *N*-acetylcysteine (NAC), 1 mM *N*-benzylhydroxylamine (N-BHA), 4 mM pyruvate or 10^4 units/mL catalase for 3 days in the presence of 5 µM acrolein or 5 µM H_2O_2. The number of bile canalicular lumen were counted and expressed as numbers of lumen/100 cells. Values are expressed as mean + SD of three independent experiments. *, $p < 0.05$, **, $p < 0.01$ against None.

Supplementaly Figure S6

Fig. 5

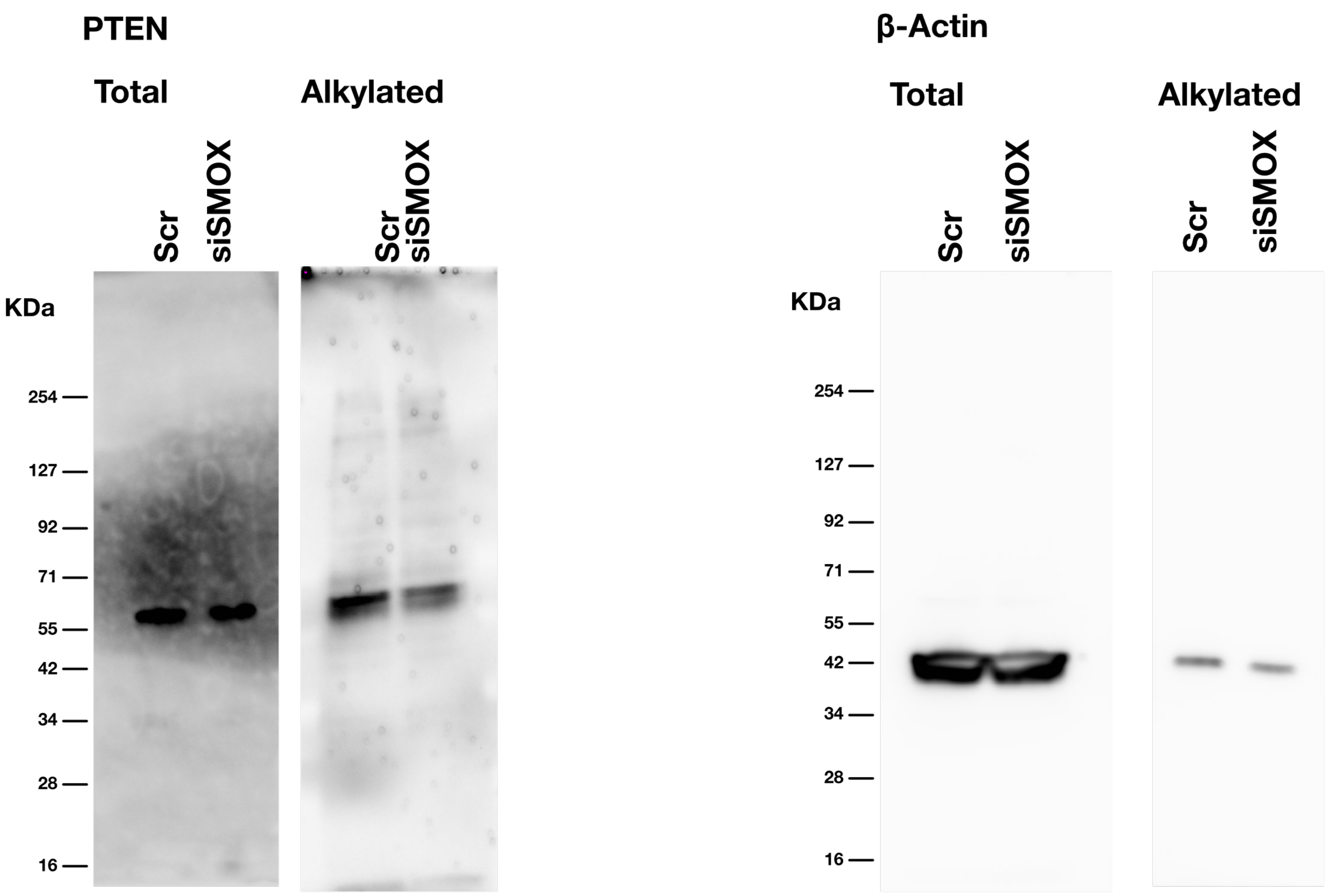

Supplementary Figure S6. Full-length blots/gels for Fig. 5 are presented. Three million cells were transfected with Scr and siSMOX RNAs, cultured for 3 days. Alkylated PTEN and β-actin were biotin-tagged and detected as described in Materials and methods section. Total PTEN and β-actin levels were measured by western blot analysis using 40 μg of cell extract.
